# Demonstrating the Non-inferiority of a Plastic Surgery Microscopic Technique for Hepatic Artery Anastomosis in Pediatric Liver Transplant: A Single-Institution Study

**DOI:** 10.7759/cureus.67742

**Published:** 2024-08-25

**Authors:** Emily L Isch, Emily Yanoshak, David Ebbott, Theodore E Habarth-Morales, Mario Aycart, Edward J Caterson

**Affiliations:** 1 Department of General Surgery, Division of Plastic Surgery, Thomas Jefferson University Hospital, Philadelphia, USA; 2 Department of Plastic Surgery, Thomas Jefferson University, Philadelphia, USA; 3 Department of Plastic Surgery, Nemours Children's Hospital, Wilmington, USA

**Keywords:** hepatic artery thrombosis, donor criteria, graft survival, loupe-assisted anastomosis, microsurgical anastomosis, pediatric liver transplantation

## Abstract

Introduction: Before advancements in liver transplantation, conditions such as acute liver failure, decompensated liver cirrhosis, and hepatocellular carcinoma were associated with poor prognosis. Orthotopic liver transplantation has since emerged as a curative treatment. Despite its benefits, liver transplantation can lead to complications, including hepatic artery thrombosis (HAT), which is especially significant in pediatric patients. This study evaluates the utility of microsurgical anastomosis by plastic surgeons in reducing postoperative HAT compared to standard loupe-assisted anastomosis performed by transplant surgeons.

Methods: This retrospective chart review included pediatric patients who underwent orthotopic liver transplantation at a single institution between September 2015 and September 2023. Patients were divided into two groups: one receiving standard loupe-assisted anastomosis by transplant surgeons (n = 28) and the other receiving microsurgical anastomosis by plastic surgeons (n = 22). The primary outcomes measured were the rates of HAT. Secondary outcomes included graft survival, patient survival, postoperative hospital stay, resistive indices, bleeding, biliary complications, venous complications, transplant rejection, and reoperation rates.

Results: In 50 pediatric patients who underwent orthotopic liver transplantation, we compared outcomes between standard anastomosis (n = 28) and microscope-assisted anastomosis (n = 22). Demographic characteristics were similar between the groups. Hemorrhage occurred significantly more frequently in the standard anastomosis group (35.7%) compared to the microscope-assisted group (9.1%), with a p-value of 0.045. Other complications, including HAT (28.6% vs. 13.6%, p = 0.306), biliary leak (14.3% vs. 27.3%, p = 0.302), and organ rejection (21.4% vs. 13.6%, p = 0.713), did not differ significantly between the groups. Additionally, survival rates were comparable, with 71% in the standard group and 86% in the microscope group (p = 0.306). These findings suggest that while microscope-assisted anastomosis may reduce the risk of hemorrhage, other outcomes remain similar between the techniques.

Conclusion: Our findings suggest that microsurgical anastomosis techniques performed by plastic surgeons are non-inferior to standard loupe-assisted techniques in pediatric liver transplantation and may decrease the rate of postoperative hemorrhage. Microsurgical anastomosis is a viable alternative to standard loupe-assisted techniques in pediatric hepatic artery transplants. Further research with larger sample sizes is warranted to confirm these findings and optimize surgical techniques.

## Introduction

Before advancements in liver transplantation, patients diagnosed with acute liver failure, decompensated liver cirrhosis, and hepatocellular carcinoma faced a poor prognosis [1,2]. Orthotopic liver transplantation has since emerged as a curative treatment option for these conditions [1]. Although deceased donor transplantation remains a common approach, recent advancements, such as machine perfusion, have improved the salvage of living donor liver transplants [3].

The common indications for liver transplant are different in children than adults, with cholestatic biliary atresia being the leading indication for liver transplantation in the pediatric population [4]. This may stem from a delayed diagnosis of biliary atresia, failed Kasai portoenterostomy, recurrent cholangitis, and advancing portal hypertension [5]. At approximately 500 cases a year, pediatric liver transplants account for 7-8% of total liver transplants in the United States, with most transplants occurring at less than two years of age [6,7]. Recent outcomes of pediatric liver transplantation show survival rates at one and five years to be 91-94% and 84-86.5%, respectively, indicating liver transplantation to be an effective treatment option for liver failure [5].

Despite its benefits, liver transplantation can lead to complications, including vascular ones like hepatic artery thrombosis (HAT). HAT is a common and potentially devastating complication, and ensuring successful hepatic artery anastomosis is crucial due to its role in supplying oxygenated blood to the biliary system [8]. HAT can cause bile duct ischemia, often necessitating re-transplantation and significantly increasing mortality rates [9]. In the pediatric population, HAT is a significant cause of graft loss after liver transplant, with studies citing an incidence rate of 5.7% to 8.4% [5].

Several studies have focused on HAT, identifying factors that mitigate its complications. These include early HAT detection using twice-daily Doppler, increased surgical experience, and refinements in surgical technique [10,11]. Notably, one study found a decreased incidence of HAT with microsurgical techniques, use of living donors, and involving plastic surgeons trained in microsurgery for hepatic artery anastomosis [12].

In this quality improvement initiative, we examined data from one institution to evaluate the utility of plastic surgery microvascular anastomosis in minimizing postoperative HAT in pediatric patients. We hypothesized that microsurgical techniques could improve HAT and patient survival rates and potentially increase the range of organs able to be transplanted, including organs with smaller caliber vessels difficult to anastomose using standard techniques. Our study compared HAT rates when anastomoses were performed by a plastic surgeon using microsurgical techniques and a transplant surgeon using standard techniques.

## Materials and methods

Study design and patient population

This retrospective cohort study was conducted at Nemours Children’s Hospital, involving pediatric patients who underwent orthotopic liver transplantation (OLT) between September 2015 and September 2023. The study was exempt from institutional review board approval as this was a quality improvement effort. Informed consent was waived due to the retrospective nature of the study. The patient population consisted of 50 children, divided into two groups based on the surgical technique used for hepatic artery anastomosis: the standard anastomosis group (n = 28) and the microscope-assisted anastomosis group (n = 22).

Inclusion and exclusion criteria

Patients were included in the study if they were under 18 years of age at the time of transplantation and had undergone a primary OLT. Patients with previous liver transplants, multi-organ transplants, or those with incomplete medical records were excluded from the analysis.

Surgical techniques

In the standard anastomosis group, hepatic artery anastomosis was performed using loupe magnification with standard microsurgical instruments. The microscope-assisted group underwent hepatic artery anastomosis using a high-powered surgical microscope, allowing for enhanced visualization of the vascular structures. The standard anastomosis technique was performed by experienced transplant surgeons using loupe magnification, and the microscopic anastomosis technique was performed by experienced plastic surgeons, all of whom followed standardized protocols.

Data collection

Patient demographic data, including age, gender, ethnicity, and race, were collected from electronic medical records. Clinical data, including intraoperative details, postoperative complications, and survival outcomes, were also retrieved. Complications of interest included hemorrhage, HAT, biliary leak, biloma, organ rejection, bile duct stricture, intra-abdominal abscess, pericardial effusion, chylous ascites, post-transplant lymphoproliferative disorder (PTLD), cholangitis, enterocutaneous fistula, disseminated adenovirus, and thoracic duct obstruction. All complications were categorized according to standardized criteria and were documented if they occurred within one year of the transplantation.

Statistical analysis

Descriptive statistics were calculated for all demographic and clinical variables. Categorical variables were compared between the standard anastomosis and microscope-assisted anastomosis groups using Fisher’s exact test. The primary outcome measure was the incidence of HAT, while secondary outcomes included the rates of other complications and overall survival. Statistical significance was determined at a p-value of <0.05. Data analysis was conducted using SPSS version 29 (IBM Corp., Armonk, NY). All patient data were anonymized to ensure confidentiality.

## Results

Our study was a retrospective analysis of 50 pediatric patients who underwent orthotopic liver transplants between September 2015 and September 2023. The study had 28 patients in the standard anastomosis group and 22 in the microscope group. Demographics of the study participants are included in Table 1. There were no statistical differences between the groups. These demographic characteristics illustrate the similarity between the two groups, allowing for a meaningful comparison of clinical outcomes associated with the different anastomosis techniques.

**Table 1:** Demographic information for loupe versus microscope-assisted anastomosis populations. Statistical significance was determined when p < 0.05.

	Standard anastomosis (N, %)	Microscope-assisted anastomosis (N, %)	Fisher's exact test, P-value
	N = 28	N = 22	
Mean age at transplantation	4.69 years	4.10 years	-
Gender			
Male	8 (28.57%)	7 (31.82%)	1
Female	20 (71.43%)	15 (68.18%)	1
Ethnicity			
Non-Hispanic or Latino	19 (67.86%)	15 (68.18%)	1
Another Hispanic, Latino, or Spanish origin	5 (17.86%)	6 (27.27%)	0.503
Puerto Rican	1 (3.57%)	1 (4.55%)	1
Mexican, Mexican American, Chicano	2 (7.14%)	0 (0.00%)	0.497
Race			
Some other race	6 (21.43%)	7 (31.82%)	0.52
Black or African American	8 (28.57%)	5 (22.73%)	0.751
White or Caucasian	11 (39.29%)	9 (40.91%)	1
Asian Indian	0 (0.00%)	1 (4.55%)	0.44
Some other race, White or Caucasian	1 (3.57%)	0 (0.00%)	1
Black or African American, White or Caucasian	0 (0.00%)	1 (4.55%)	0.44
Black or African American, some other race	1 (3.57%)	1 (4.55%)	1

As seen in Table 2, the primary outcome of HAT did differ between the standard versus microscope-assisted group (18% vs. 9%) but did not reach statistical significance with a p-value of 0.444 determined by Fisher’s exact test.

**Table 2:** Comparing rates of hepatic artery thrombosis in standard vs. microscope-assisted anastomoses. The data have been represented as numbers. Statistical significance was determined when p < 0.05. HAT: hepatic artery thrombosis.

	HAT	No HAT	Total	Fisher's exact test, P-value
Standard	5	23	28	0.444
Microscope	2	20	22	
Total	7	43	50	

In comparing complications between patients who underwent standard anastomosis (n = 28) and those who underwent microscope-assisted anastomosis (n = 22), hemorrhage was significantly more common in the standard anastomosis group (35.7%) compared to the microscope-assisted group (9.1%), with a p-value of 0.045. Other complications, including HAT (28.6% vs. 13.6%, p = 0.306), biliary leak (14.3% vs. 27.3%, p = 0.302), organ rejection (21.4% vs. 13.6%, p = 0.713), and chylous ascites (7.1% vs. 18.2%, p = 0.385), did not show statistically significant differences between the groups. Additionally, some complications such as bile duct stricture, intra-abdominal abscess, and pericardial effusion showed no significant differences, with p-values ranging from 0.44 to 1.0. These findings, demonstrated in Table 3, suggest that while hemorrhage was significantly reduced with microscope-assisted anastomosis, other complications occurred at similar rates between the two groups.

**Table 3:** Comparison of complication rates between standard and microscope anastomoses. The data have been represented as percentages. Statistical significance was determined when p < 0.05.

Complication	Standard anastomosis (N = 28)	Percentage (%)	Microscope anastomosis (N = 22)	Percentage (%)	Fisher's exact test, P-value
Hemorrhage	10	35.70%	2	9.10%	0.045
Hepatic artery thrombosis (HAT)	8	28.60%	3	13.60%	0.306
Biliary leak	4	14.30%	6	27.30%	0.302
Biloma	2	7.10%	1	4.50%	1
Organ rejection	6	21.40%	3	13.60%	0.713
Bile duct stricture	2	7.10%	-	-	0.497
Intra-abdominal abscess	1	3.60%	-	-	1
Pericardial effusion	1	3.60%	2	9.10%	0.576
Chylous ascites	2	7.10%	4	18.20%	0.385
Post-transplant lymphoproliferative disorder (PTLD)	1	3.60%	-	-	1
Cholangitis	1	3.60%	-	-	1
Enterocutaneous fistula	-	-	1	4.50%	0.44
Disseminated adenovirus	-	-	1	4.50%	0.44
Thoracic duct obstruction	-	-	1	4.50%	0.44

Rates of survival did not significantly differ between the two groups; 71% (20 out of 28) in the standard group, and 86% (19 out of 22) in the microscope group; with a p-value of 0.306, there was no statistical difference between the groups, as seen in Table 4.

**Table 4:** Chi-square test for survival rates comparing standard versus microscope anastomoses. The data have been represented as numbers. Statistical significance was determined when p < 0.05.

	Survivors	Non-survivors	Total	Fisher's exact test, P-value
Standard	20	8	28	0.306
Microscope	19	3	22	
Total	39	11	50	

## Discussion

Our quality improvement initiative investigated the use of microsurgical anastomosis to reduce postoperative HAT compared to standard anastomotic techniques. Microsurgery, involving operating microscopes and specialized instruments like loupes, allows for the anastomosis of smaller blood vessels and nerves. This technique has been pivotal in surgeries involving free grafting of tissue [13]. Proper visualization of the surgical field is essential in microsurgery, as poor visualization may lead to damage to nearby structures, which can affect surgical outcomes, reduce organ preservation, or even cause life-threatening consequences [14].

A surgical microscope, also known as an operating microscope, is an optical microscope specifically designed for surgery, offering magnification from six to 40 times greater than that of the unaided human eye [15]. Operating microscopes have high-precision optics and high-power coaxial illumination. These features provide surgeons with adjustable magnifications, proper working distance, and an unobstructed view of the surgical field. This system offers stability and maneuverability and improves ergonomics [16].

Alternatively, surgical loupes magnify objects from two to eight times that of the unaided eye. Compared to microscopes, loupes have advantages regarding cost, flexibility, portability, and time savings. Other qualities of loupes include closer access to the surgical field, wider orientation, rapid changes in viewing angle, depth of field, and adjustment of gaze location in the surgical field via changes in the surgeon’s head and neck [15].

Many studies have been conducted examining the outcomes of instruments used for microsurgery. Previous studies comparing the use of loupes versus the operating microscope have shown no statistically significant differences in the outcomes of free flaps, minimally invasive transforaminal lumbar interbody fusion surgeries, and endodontic surgeries [17-19].

In hepatic artery anastomosis, microscopes aid not only in vessel anastomosis but also in identifying other HAT risk factors, like uneven intima edges or thrombi in arterial stumps [11]. However, even the highest magnification with loupe-assisted anastomoses (4.5x) falls short compared to the 9x lens on a microscope.

This study provides a comprehensive comparison of clinical outcomes between standard anastomosis and microscope-assisted anastomosis in pediatric liver transplantation. While demographic characteristics were similar between the two groups, the analysis revealed a significant reduction in hemorrhage rates in the microscope-assisted anastomosis group, indicating a potential advantage of this technique in minimizing intraoperative and postoperative bleeding. However, other complications, including HAT, biliary leak, organ rejection, and chylous ascites, did not show statistically significant differences between the groups. Furthermore, survival rates were comparable, with no significant difference observed between the two techniques.

This study had limitations, including insufficient data for meaningful analysis of certain diagnostic criteria for transplantation. Nonetheless, our findings suggest that microsurgical techniques, when performed by experienced plastic surgeons, have the potential to reduce postoperative hemorrhage, and produce non-inferior rates of HAT in pediatric liver transplants. This anastomosis technique should be seen as a viable option for utilizing donor organs with challenging vascular anatomy.

## Conclusions

Our findings suggest that while microscope-assisted anastomosis may offer some benefits in reducing specific complications such as hemorrhage, both techniques yield similar overall clinical outcomes in pediatric liver transplantation. Further studies with larger cohorts and long-term follow-up are warranted to validate these findings and explore the potential advantages of microscope-assisted anastomosis in more detail.

## References

[1] Graziadei I, Zoller H, Fickert P (2016). Indications for liver transplantation in adults: recommendations of the Austrian Society for Gastroenterology and Hepatology (ÖGGH) in cooperation with the Austrian Society for Transplantation, Transfusion and Genetics (ATX). Wien Klin Wochenschr.

[2] Martin P, DiMartini A, Feng S, Brown R Jr, Fallon M (2014). Evaluation for liver transplantation in adults: 2013 practice guideline by the American Association for the Study of Liver Diseases and the American Society of Transplantation. Hepatology.

[3] Aziz H, Nayak P, Mulligan DC (2024). Current status of liver transplantation in North America. Surg Clin North Am.

[4] Smith SK, Miloh T (2022). Pediatric liver transplantation. Clin Liver Dis.

[5] Pham YH, Miloh T (2018). Liver transplantation in children. Clin Liver Dis.

[6] Rawal N, Yazigi N (2017). Pediatric liver transplantation. Pediatr Clin North Am.

[7] Kwong AJ, Ebel NH, Kim WR (2023). OPTN/SRTR 2021 annual data report: liver. Am J Transplant.

[8] Amara D, Parekh J, Sudan D (2022). Surgical complications after living and deceased donor liver transplant: the NSQIP transplant experience. Clin Transplant.

[9] Busuttil RW, Goss JA (1999). Split liver transplantation. Ann Surg.

[10] Pamecha V, Sasturkar SV, Sinha PK, Mohapatra N, Patil N (2021). Biliary reconstruction in adult living donor liver transplantation: the all-knots-outside technique. Liver Transpl.

[11] Lee CF, Lu JC, Zidan A, Lee CS, Wu TH, Chan KM, Lee WC (2017). Microscope-assisted hepatic artery reconstruction in adult living donor liver transplantation—a review of 325 consecutive cases in a single center. Clin Transplant.

[12] Trabelsi NO, Melhem HB, Matouk MA, Borsuk DE, Efanov JI (2023). Rates of hepatic artery thrombosis in liver transplantation with the use of a microscope: a systematic review. J Plast Reconstr Aesthet Surg.

[13] Mavrogenis AF, Markatos K, Saranteas T (2019). The history of microsurgery. Eur J Orthop Surg Traumatol.

[14] Ross DA, Ariyan S, Restifo R, Sasaki CT (2003). Use of the operating microscope and loupes for head and neck free microvascular tissue transfer: a retrospective comparison. Arch Otolaryngol Head Neck Surg.

[15] Stanbury SJ, Elfar J (2011). The use of surgical loupes in microsurgery. J Hand Surg Am.

[16] Ma L, Fei B (2021). Comprehensive review of surgical microscopes: technology development and medical applications. J Biomed Opt.

[17] Ehanire T, Singhal D, Mast B, Leyngold M (2018). Safety of microsurgery under loupes versus microscope: a head-to-head comparison of 2 surgeons with similar experiences. Ann Plast Surg.

[18] Singhatanadgige W, Chamadol H, Tanasansomboon T, Kang DG, Yingsakmongkol W, Limthongkul W (2022). Surgical outcomes of minimally invasive transforaminal lumbar interbody fusion using surgical microscope vs surgical loupes: a comparative study. Int J Spine Surg.

[19] Taschieri S, Weinstein T, Tsesis I, Bortolin M, Del Fabbro M (2013). Magnifying loupes versus surgical microscope in endodontic surgery: a four-year retrospective study. Aust Endod J.

